# Association between triglyceride glucose index and adverse clinical outcomes in patients with acute myocardial infarction and LDL-C≤1.8 mmol/L who underwent percutaneous coronary intervention: a prospective cohort study

**DOI:** 10.3389/fendo.2023.1323615

**Published:** 2024-01-18

**Authors:** Hong-wei Zhao, Yong Wang, Cheng-fu Wang, Qing-kun Meng

**Affiliations:** ^1^ Department of Cardiology, The Seventh Affiliated Hospital of Sun Yat-sen University, Shenzhen, China; ^2^ Department of Cardiology, Shenzhen Luohu Hospital Group Luohu People’s Hospital (The Third Affiliated Hospital of Shenzhen University), Shenzhen, China; ^3^ Department of Cardiology, The People’s Hospital of Liaoning Province, Shenyang, China

**Keywords:** triglyceride glucose index, major adverse cardiac and cerebrovascular event, acute myocardial infarction, low density lipoprotein, PCI

## Abstract

**Background:**

Recently, the triglyceride glucose (TyG) index has emerged as a reliable predictive indicator for adverse outcomes of cardiovascular disease. However, the roles of the TyG index in patients with acute myocardial infarction (AMI) and low-density lipoprotein cholesterol (LDL-C)≤1.8 mmol/L after percutaneous coronary intervention (PCI) remain unclear.

**Methods:**

A total of 599 patients diagnosed with AMI and LDL-C ≤ 1.8 mmol/L at the 1-month follow-up after PCI were consecutively enrolled between January 2017 and January 2020. The patients were subsequently divided into three groups based on tertiles of the TyG index. The parameters, including the TyG index, were compared to explore the risk factors associated with major adverse cardiovascular and cerebrovascular events (MACCEs) during the 1-year follow-up.

**Results:**

Sixty-nine patients (11.5%) with 90 MACCEs were recorded during the 1-year follow up, including 13 patients (8.6%) in the Tertile 1 group, 36 (12.0%) in the Tertile 2 group, and 20 (13.4%) in the Tertile 3 group. Patients with a higher TyG index had a significantly increased incidence of MACCEs compared to those with a lower TyG index (22.1% vs. 14.0% vs. 9.9%, p=0.010). Kaplan–Meier analysis demonstrated that patients with a higher TyG index had a significantly lower probability of survival without MACCEs. Furthermore, a binary logistic regression model indicated that the TyG index was the only independent predictor for MACCEs in these patients.

**Conclusion:**

A higher TyG index was associated with a higher incidence of MACCEs in patients with AMI and well-controlled LDL-C levels after PCI. This suggests that the TyG index can serve as a predictive indicator for adverse cardiovascular outcomes in these patients.

## 1 Introduction

Acute myocardial infarction (AMI) is the leading cause of morbidity and mortality in people with cardiovascular disease (CVD) worldwide, accounting for approximately one million deaths annually in China (1, 2). With the development of percutaneous coronary intervention (PCI) techniques, well-controlled traditional cardiovascular risk factors, and optimized guideline-directed medical therapy, the morbidity and mortality of AMI have been significantly reduced. Low-density lipoprotein cholesterol (LDL-C) is a well-established risk factor for CVD, and various guidelines and clinical trials have emphasized LDL-C target levels. In recent years, most guidelines have recommended a target LDL-C level of ≤1.8 mmol/L for patients with established CVD and a more aggressive target LDL-C level of ≤1.4 mmol/L for patients at high risk (3, 4). Despite optimal management of LDL-C (≤ 1.8 mmol/L), a significant number of patients continue to suffer adverse outcomes (5). Therefore, it is of utmost importance to investigate residual risk factors for the development of future major adverse cardiovascular and cerebrovascular events (MACCEs) to improve the management of these patients.

Insulin resistance (IR) plays a crucial role in the development of diabetes mellitus and has been suggested as a potential risk factor for CVD (6). Indeed, individuals with IR are at a higher risk of developing various metabolic disorders, including hyperglycemia, dyslipidemia, and hypertension, which are strongly related to poor outcomes in CVD (7). However, in clinical practice, there are no specific, direct, convenient, and accurate methods for the assessment of IR. Therefore, the triglyceride glucose (TyG) index has been suggested as a reliable surrogate biomarker for IR (8). Previous studies have consistently demonstrated that elevated levels of the TyG index are an independent predictor for increased arterial stiffness (9), as well as the presence of coronary and carotid atherosclerosis (10, 11). Furthermore, emerging evidence suggests that a higher TyG index is associated with poor outcomes in patients with acute coronary syndrome (ACS) with and without diabetes mellitus who undergo PCI (12, 13). Additionally, an elevated TyG index has been linked to an increased risk of AMI (14), as well as repeat revascularization and in-stent restenosis in patients with chronic coronary syndrome who undergo PCI (15). However, the potential association between the TyG index and MACCEs in patients with AMI and well-controlled LDL-C at 1-month follow-up after PCI remains unclear. Therefore, we aimed to explore possible relationships between the TyG index and MACCEs in these patients.

## 2 Methods

### 2.1 Study population

**Figure 1:**
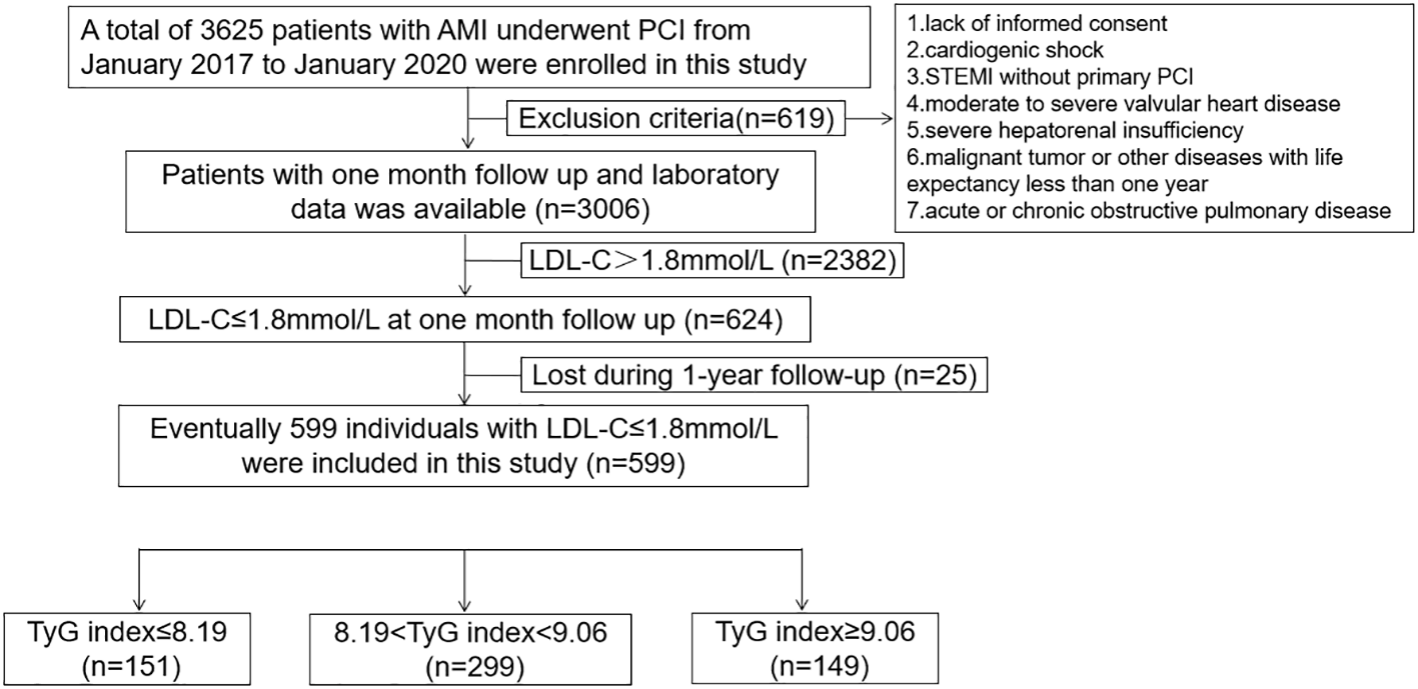
Study flow chart.

This is a prospective cohort study, which consecutively included 3625 patients with AMI who underwent PCI from January 2017 to January 2020. The flow chart is displayed in **Figure 1**. The exclusion criteria were as follows: 1) lack of informed consent; 2) cardiogenic shock; 3) ST-elevated myocardial infarction (STEMI) without primary PCI; 4) moderate to severe valvular heart disease; 5) severe hepatorenal insufficiency; 6) malignant tumor or other diseases with life expectancy less than one year; and 7) acute or chronic obstructive pulmonary disease. In total, 619 patients were excluded from this study. All the patients received a follow-up every month after discharge. Patients with LDL-C>1.8 mmol/L were excluded from this study at the 1-month follow-up after PCI. Eventually, 599 patients with AMI and LDL-C ≤ 1.8 mmol/L who had undergone PCI during the 1-month follow-up were enrolled in this study. The individuals included were then divided into three groups according to TyG index tertiles. The parameters were compared, and MACCEs were recorded during the 1-year follow up. All the patients with STEMI received a primary PCI according to the guidelines (16). The study was conducted according to the principles of the Declaration of Helsinki. All patients provided informed consent prior to their participation in the study.

### 2.2 Clinical and laboratory data assessments and definitions

After hospitalization, the baseline characteristics, medical history, clinical presentation, target lesions, lesion characteristics, laboratory parameters, and medications received in the hospital were recorded. All blood samples were collected from the median cubital vein after overnight fasting. All the laboratory parameters were collected prospectively using a standardized data collection form. The angiographic data and lesion characteristics were obtained from the cardiac catheterization laboratory records. Follow-up was performed via outpatient clinic, readmission, or phone contact. The TyG index was calculated as Ln (fasting triglycerides (TG) (mg/dL)×fasting blood glucose (mg/dL)/2) (8).

### 2.3 Follow−up and endpoints

The primary outcome was MACCEs, defined as a composite of all-cause death, target lesion revascularization (TLR), target vessel revascularization (TVR), AMI, and ischemic stroke. Deaths were considered cardiac causes unless a definitive noncardiac cause was found (17). AMI was diagnosed according to the Fourth Universal Definition of Myocardial Infarction (18). TLR was defined as the revascularization of restenosis in stent or less than 5 mm from the proximal or distal margins (19). TVR was defined as revascularization of any culprit vessel or its main branches (19). All the patients received follow-up monthly after discharge until an end point occurred or the 1-year follow-up period was completed.

### 2.4 Statistical analysis

Statistical analyses were conducted using SPSS version 20.0 (IBM, USA). All the patients included were grouped into tertiles according to the TyG index: Tertile 1 (n=151, TyG index ≤ 8.19), Tertile 2 (n=299, 8.19<TyG index<9.06), and Tertile 3 (n=149, TyG index≥9.06), and the characteristics were compared among the three groups. Categorical variables are displayed as rates or percentages, which were analyzed using chi-square or Fisher’s exact tests. Continuous variables with normal and non-normal distributions are presented as the mean ± standard deviation and median (interquartile range), respectively. These data were analyzed using one-way analysis of variance and the Kruskal–Wallis test, respectively. Univariate analysis was conducted to investigate the potential risk predictors for MACCEs in patients with AMI who underwent PCI. Then, five multivariate variables were included to explore the independent predictors for MACCEs in these patients. Receiver operating characteristic (ROC) curve analysis was conducted to evaluate the predictive value of the TyG index for the incidence of MACCEs in patients with AMI and LDL-C ≤ 1.8 mmol/L who underwent PCI. Kaplan–Meier curves and log-rank tests were performed to compare the MACCE-free survival among the three groups. All tests were two-sided, and p-values<0.05 were considered significant.

## 3 Results

### 3.1 Baseline and clinical characteristics

A total of 599 patients with AMI and LDL-C ≤ 1.8 mmol/L at 1-month follow-up were enrolled in this study (**Figure 1**). Baseline characteristics, medical history, clinical presentation, target lesions, lesion characteristics, laboratory parameters, and medications received in the hospital are shown in **Table 1**. The patients included in this study were grouped according to the TyG index (≤8.19, 8.19–9.06, and ≥9.06). The median TyG index in the included patients was 8.56. There were no differences between the three groups regarding age, sex, current smoking status, hypertension, heart failure, previous stroke, previous myocardial infarction, previous PCI, family history of coronary artery disease, and peripheral vascular disease (p>0.05). The clinical presentation, target lesions, lesion characteristics, and medications were also comparable among the three groups (p>0.05). However, patients with a higher TyG index at 1-month follow-up had a higher body mass index (BMI) and diabetes mellitus, as well as higher fasting blood glucose, total cholesterol, TG, and uric acid levels; a higher TyG index was associated with a lower high-density lipoprotein cholesterol (HDL-C) level (p<0.05).

**Table 1:** Baseline characteristics of the study population by tertiles of TyG-BMI index.

Variables	Tertile 1	Tertile 2	Tertile 3	P-value
Demographics				
Age, years	60.0(52.0,71.0)	60.0(52.0,69.0)	60.0(50.0,68.0)	0.488
Gender(male), n(%)	102(67.5)	186(68.9)	94(63.1)	0.463
BMI	22.23(19.29,24.51)	23.4(20.1,25.9)	25.3(23.3,26.9)	<0.001
Medical history				
Current smoker, (%)	39(25.8)	89(29.8)	40(26.8)	0.634
Diabetes Mellitus, (%)	19(12.6)	64(21.4)	60(40.3)	<0.001
Hypertension, (%)	65(43.0)	154(51.5)	80(53.7)	0.135
Heart failure, (%)	26(17.2)	33(11.0)	23(15.4)	0.153
Previous Stroke, (%)	8(5.3)	19(6.4)	9(6.0)	0.905
Previous MI, (%)	10(6.6)	12(4.0)	8(5.4)	0.475
Previous PCI, (%)	10(6.6)	30(10.0)	7(4.7)	0.115
Family history of CAD, (%)	65(43.0)	128(42.8)	59(39.6)	0.779
Peripheral vascular disease, (%)	3(2.0)	7(2.3)	4(2.7)	0.923
Clinical presentation				
NSTEMI, (%)	106(70.2)	202(67.6)	108(72.5)	0.552
STEMI, (%)	45(29.8)	97(32.4)	41(27.5)	0.552
Target lesions and Lesions characteristics				
LM, (%)	3(2.0)	10(3.3)	5(3.4)	0.919
LAD, (%)	63(41.7)	119(39.8)	59(39.6)	
LCX, (%)	39(25.8)	72(24.1)	32(21.5)	
RCA, (%)	46(30.5)	98(32.8)	53(35.6)	
1-vessel disease, (%)	69(45.7)	128(42.8)	58(38.9)	0.818
2-vessel disease, (%)	49(32.5)	105(35.1)	54(36.2)	
3-vessel disease, (%)	33(21.9)	66(22.1)	37(24.8)	
Ostio lesions, (%)	22(14.6)	36(12.0)	15(10.1)	0.489
Bifurcation lesions, (%)	34(22.5)	57(19.1)	24(16.1)	0.369
CTO lesions, (%)	0	2(0.7)	1(0.7)	0.417
Laboratory parameters				
FBG, mmol/L	4.7(4.3,5.0)	5.2(4.6,5.9)	6.3(5.1,8.6)	<0.001
TC, mmol/L	2.9(2.6,3.4)	3.0(2.7,3.3)	3.3(3.0,3.7)	<0.001
TG, mmol/L	0.7(0.6,0.9)	1.3(1.1,1.5)	2.5(1.9,3.3)	<0.001
LDL-C, mmol/L	1.5(1.2,1.7)	1.5(1.3,1.7)	1.6(1.3,1.7)	0.427
HDL-C, mmol/L	1.2(1.0,1.5)	1.0(0.8,1.3)	0.9(0.8,1.1)	<0.001
Uric acid, umol/L	270.0(230.0,332.0)	291.0(237.0,342.0)	303.0(238.0,365.0)	0.034
NT-proBNP, pg/ml	1285.3(825.3,1823.5)	1332.4(769.2,1925.4)	1298.7(802.3,2015.4)	0.825
Cr, mmol/L	69.0(57.0,83.0)	71.0(59.0,82.0)	70.0(60.0,80.0)	0.901
TyG index	7.9(7.7,8.1)	8.6(8.4,8.8)	9.5(9.2,9.7)	<0.001
Medication				
Aspirin, (%)	150(99.3)	298(99.7)	145(97.3)	0.085
Clopidogrel/Ticagrelor, (%)	151(100.0)	299(100.0)	149(100.0)	1
ACEI/ARB/ARNI, (%)	82(54.3)	174(58.2)	77(51.7)	0.397
Beta-blocker, (%)	110(72.8)	237(79.3)	107(71.8)	0.138
Calcium canal blocker, (%)	44(29.1)	74(24.7)	45(30.2)	0.392
Statin, (%)	141(93.4)	283(94.6)	139(93.3)	0.795
Ezetimibe, (%)	10(6.6)	22(7.4)	11(7.4)	0.954

BMI, body mass index; MI, myocardial infarction; PCI, percutaneous coronary intervention; CAD, coronary artery disease; NSTEMI, Non-ST-elevation myocardial infarction; STEMI, ST-Elevation Myocardial Infarction; LM, left main; LAD, left anterior descending artery; LCX, left circumflex artery; RCA, right coronary artery; CTO, chronic total occlusion; FBG, fasting blood glucose; TC,total cholesterol; TG,Triglyceride; LDL-C: low-density lipoprotein cholesterol; HDL-C, high-density lipoprotein cholesterol; TyG index, triglyceride glucose index; ACEI, angiotensin-converting enzyme inhibitor; ARB, angiotensin II receptor blocker; ARNI, angiotensin receptor enkephalinase inhibitor.

### 3.2 Incidence of clinical outcomes in the overall study population during 1-year follow-up

**Figure 2:**
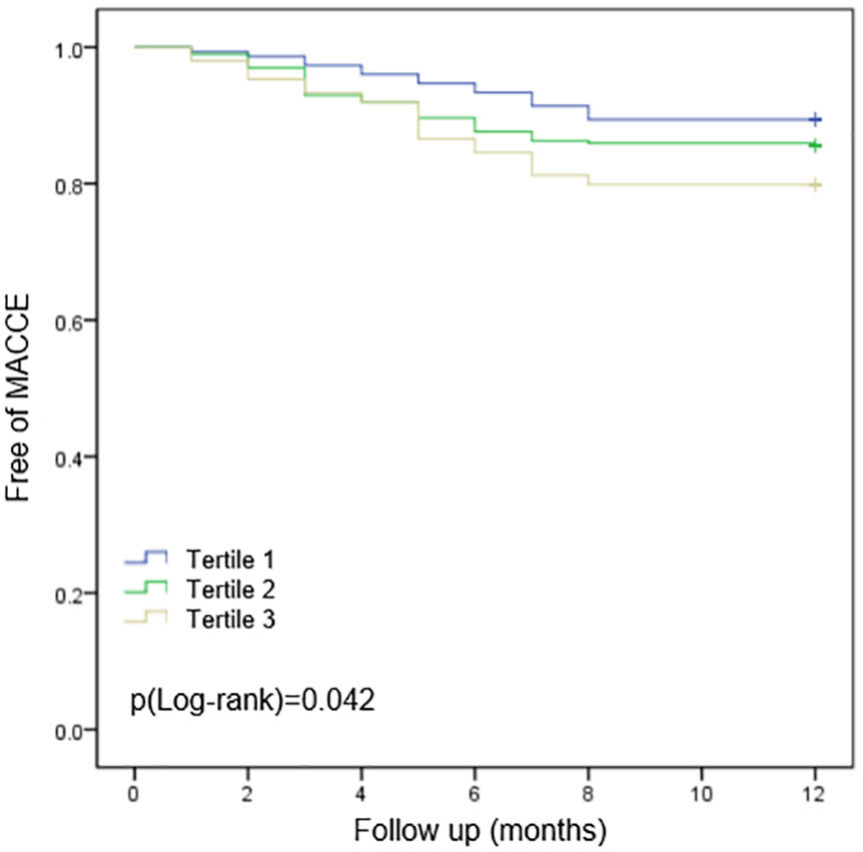
The Kaplan-Meier survival curves analysis.

All the patients were followed up to one year after PCI. The incidence of MACCEs and individual events are displayed in **Table 2**. The cumulative incidences of MACCEs were stratified according to TyG index tertiles. Sixty-nine patients (11.5%) with 90 MACCEs were recorded during the 1-year follow-up, including 13patients (8.6%) in the Tertile 1 group, 36 (12.0%) in the Tertile 2 group, and 20 (13.4%) in the Tertile 3 group. The incidences of all-cause mortality, TLR, and stroke showed no significant differences among the three groups. However, compared to those with a lower TyG index, patients with a higher TyG index showed increased incidence of TVR (10.1% vs. 4.7% vs. 2.0%, in the three tertile groups, respectively, p=0.005), AMI (5.4% vs. 1.7% vs. 1.3%, p=0.035), and MACCEs in total (22.1% vs. 14.0% vs. 9.9%, p=0.010). Kaplan–Meier analysis showed that patients with a higher TyG index had a poor clinical outcome of MACCE-free survival, which was statistically significant (**Figure 2**).

**Table 2:** Incidence of clinical outcomes in the overall population during follow-up.

Variables	Tertile 1	Tertile 2	Tertile 3	P-value
All cause death, (%)	2(1.3)	5(1.7)	5(3.4)	0.385
TLR, (%)	7(4.6)	16(5.4)	12(8.1)	0.398
TVR, (%)	3(2.0)	14(4.7)	6(10.1)	0.005
AMI, (%)	2(1.3)	5(1.7)	8(5.4)	0.035
Stroke, (%)	1(0.7)	2(0.7)	2(1.3)	0.734
MACCEs, (%),	15(9.9)	42(14.0)	33(22.1)	0.010

TLR, target lesion revascularization; TVR, target vessel revascularization; AMI, acute myocardial infarction; MACCE, major adverse cardiovascular and cerebrovascular events.

### 3.3 Association of factors with MACCEs

**Figure 3:**
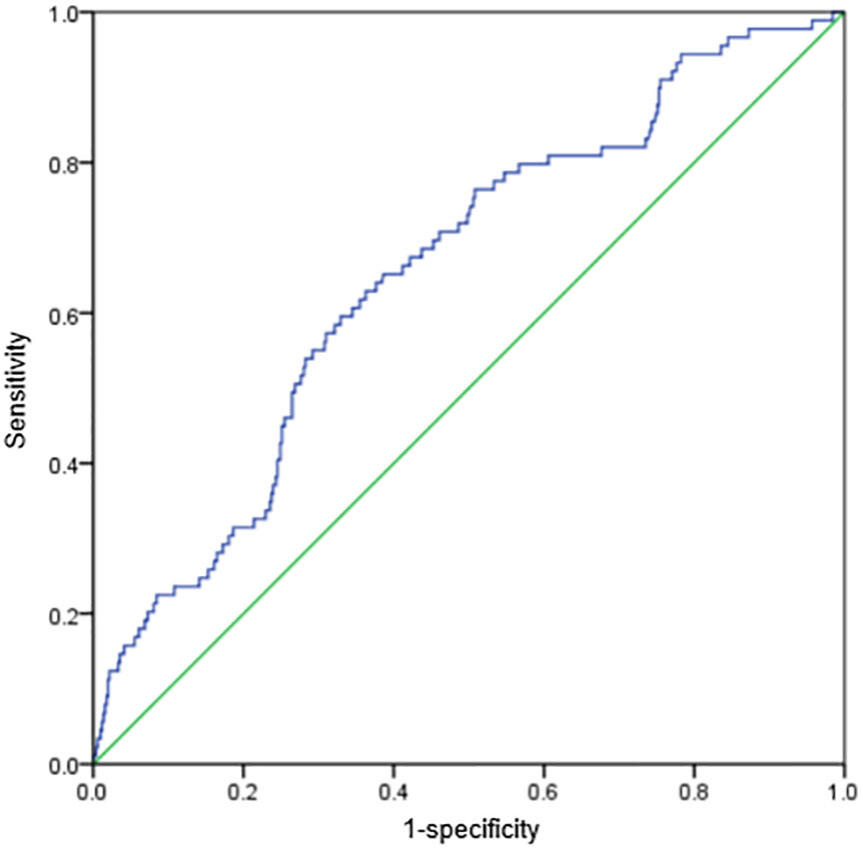
ROC curve showing the distinguishing ability of TyG index for the presence of MACCE.

Univariate analysis showed that BMI, diabetes mellitus, TyG index, TC, HDL-C, and uric acid were potential risk predictors for MACCEs in patients with AMI and LDL-C<1.8 mmol/L who underwent PCI. The binary logistics regression model revealed that TyG index was the only independent predictor for MACCEs in these patients (**Table 3**). ROC analysis showed that when the TyG index was ≥8.81, the sensitivity and specificity were 62.1% and 68.0%, respectively, and the area under the ROC curve was 0.654 (95% confidence interval: 0.594–0.714; p<0.001) (**Figure 3**).

**Table 3:** Univariate and multivariate analysis for predictors of MACCEs.

	Univariate analysis			Multivariate analysis		
	OR	95% CI	P value	OR	95% CI	P value
BMI	1.054	1.002-1.132	0.031	1.020	0.986-1.127	0.124
Diabetes mellitus	2.156	1.037-5.325	0.025	2.025	0.779-5.239	0.268
TyG index	1.366	1.024-3.254	0.016	1.381	1.021-3.552	0.021
TC	1.102	1.028-3.384	0.019	1.019	0.827-3.378	0.337
HDL-C	0.824	0.559-0.922	0.028	0.821	0.584-1.268	0.509
Uric acid/100	1.526	1.017-2.957	0.033	1.508	0.879-2.861	0.314

BMI, body mass index; TyG index, triglyceride glucose index; TC,total cholesterol; HDL-C, high-density lipoprotein cholesterol.

## 4 Discussion

In this study, we first investigated the prognostic value of the TyG index in patients with AMI and well-controlled LDL-C levels (LDL-C ≤ 1.8 mmol/L) after PCI. Our findings revealed a significant association between the TyG index and MACCEs in these patients. Importantly, this association remained significant even after adjusting for potential confounding risk factors. Kaplan–Meier analysis further revealed that patients with a higher TyG index had a poorer clinical outcome, defined as a lower rate of survival free from MACCEs. The TyG index also showed good predictive value for the occurrence of MACCEs in these patients. These findings indicate that the TyG index may serve as a promising residual risk factor for adverse outcomes in patients with AMI who have well-controlled LDL-C levels after PCI.

As a traditional risk factor for CVD, hyperlipidemia plays a crucial role in the development and progression of CVD. Among all the lipids, LDL-C has received special attention. The REVERSAL study suggested that by reducing LDL-C levels by 50%, the progression of coronary atherosclerotic plaques could be postponed (20). A meta-analysis demonstrated that for every 1.0 mmol/L reduction in LDL-C, there was a significant 23% decrease in the risk of MACCEs (21). Currently, most related guidelines recommend a target LDL-C level of ≤1.8 mmol/L for patients with established CVD and a more aggressive LDL-C target of ≤1.4 mmol/L for patients at high risk (3, 4). However, even with optimal PCI and well-controlled cardiovascular risk factors, including LDL-C levels (≤1.4 mmol/L), a significant number of patients still suffer from MACCEs. Therefore, it is crucial to explore potential residual cardiovascular risk factors to enhance clinical practice and improve risk stratification.

The TyG index has been suggested as a reliable surrogate biomarker for IR (8), which has been shown to contribute to endothelial dysfunction, oxidative stress, and inflammatory responses (12). These adverse pathologic factors have been associated with a worse CVD prognosis (22). Additionally, IR is associated with systemic lipid disturbances, such as elevated TG and small dense LDL levels, as well as reduced levels of HDL-C, which may contribute to the progression of atherosclerosis (23). Furthermore, a reduction in insulin activity in ischemic myocardium could increase oxygen consumption due to a shift from glucose towards fatty acid metabolism, which would accelerate the progression of atherosclerosis (24). In this study, we discovered that patients with a higher TyG index suffered a higher prevalence of TVR, AMI, and MACCEs in total. Similar to previous studies, other researchers suggested that an elevated TyG index has been linked to an increased risk of AMI (14), as well as repeat revascularization and in-stent restenosis in patients with chronic coronary syndrome who have undergone PCI (15). We speculated that patients with a higher TyG index may have adverse pathologic factors (endothelial dysfunction, oxidative stress, and inflammatory responses), systemic lipid disturbances, and imbalanced oxygen consumption, which accelerate the progression of coronary atherosclerosis, resulting in poor prognosis.

Previous studies have suggested that the TyG index is closely related to various cardiovascular risk factors (25–27). Patients with elevated TyG levels may have dysglycemia, a traditional risk factor for CVD. Studies have suggested that elevated TG levels can induce increased levels of free fatty acids and promote the increased flux of free fatty acids from adipose to non-adipose tissue, which may accompany IR (28). Moreover, TG-rich-ApoB within the coronary wall is considered as an important pathogenesis of coronary atherosclerosis (29). Abnormal blood glucose and lipid metabolism are risk factors for CVD, which could lead to poor prognosis (30). In this study, patients with a higher TyG index tended to have a higher incidence of diabetes mellitus; elevated levels of blood glucose, total cholesterol, and TG; and a reduced level of HDL-C. These conditions offer another explanation for the poor prognosis in patients with an elevated TyG index.

Abnormal blood glucose levels cause damage not only to the macrovascular network but also to the microvascular network. Zhao et al. discovered that an increased TyG index was significantly related to a higher risk of arterial stiffness and microvascular damage (31). In AMI, especially in individuals with high thrombotic burden, a microvascular embolism caused by PCI could aggravate microvascular damage. Microvascular dysfunction, together with epicardial vessel stenosis or spasm are important contributors to the pathogenesis of myocardial ischemia (32). Bolognese et al. suggested that microvascular dysfunction is an important predictor of both left ventricular remodeling and unfavorable long-term outcomes for patients with AMI after primary angioplasty (33). From this perspective, patients with an elevated TyG index are more prone to suffer from microvascular damage, which was another explanation for the poor prognosis of these patients.

In this study, we found that the TyG index is associated with an increased risk of MACCEs through various mechanisms in patients with AMI and well-controlled LDL-C levels after PCI. The TyG index is considered a residual risk factor for CVD. Therefore, it is important to pay attention to TyG levels, especially in patients with well-controlled LDL-C levels. TyG could be used as a prognostic indicator as well as a factor for risk stratification.

This study had some limitations. First, this was a single-center study with a relatively small sample size. We did not collect the TyG data during follow-up periods such as 3, 6, and 12 months. Second, although multivariate analyses were performed, residual covariates may still exist, which may have affected the predictive value. Third, the patients were not grouped according to diabetes mellitus and non-diabetes mellitus status due to a relatively small sample size. Fourth, in our hospital only atorvastatin and ezetimibe were available, so all the patients were prescribed with atorvastatin and ezetimibe respectively. The dose of atorvastatin was 20mg daily and ezetimibe 10mg daily. During the follow up period, the medication was changed according to LDL-C levels. If LDL-C>1.8mmol/L, the ezetimibe was added during follow up. As PCSK9 was not available, we only use atorvastatin and ezetimibe for LDL-C lowing treatment. We didn’t use a dose of 40mg atorvastatin in this study. It is a pity, we didn’t collect the data of medication changing during follow-up, which may have an impact on the prognosis. Fifth, health-related factors such as smoking cessation, dietary patterns, and levels of daily physical activity, which may have an impact on the TyG index, were not considered in this study. This may have influenced the results. Lastly, in patients with AMI, a target LDL-C of ≤1.4 mmol/L should be considered; however, this study was performed before the latest guidelines were published. Moreover, the number of patients with LDL-C ≤ 1.4 mmol/L was limited. Future large sample, multi-center studies are needed to validate our conclusions.

## 5 Conclusion

We found that a higher TyG index was associated with a higher incidence of MACCEs in patients with AMI and well-controlled LDL-C levels after PCI. This suggests that the TyG index can serve as a predictive indicator for adverse cardiovascular outcomes in these patients.

## Data availability statement

The datasets generated and analysed during the current study are not publicly available due to a further study of this area but are available from the corresponding author on reasonable request.

## Ethics statement

The study was approved by the ethics committee of The Seventh Affiliated Hospital of Sun Yat-sen University and all the subjects provided their written informed consent before participation.

## Author contributions

H-WZ: Conceptualization, Investigation, Supervision, Validation, Writing – original draft, Writing – review & editing. YW: Conceptualization, Data curation, Formal Analysis, Methodology, Validation, Writing – original draft. C-FW: Data curation, Investigation, Methodology, Software, Validation, Writing – review & editing. Q-KM: Conceptualization, Formal Analysis, Methodology, Resources, Validation, Writing – review & editing.
